# Evaluation of DeChoker, an Airway Clearance Device (ACD) Used in Adult Choking Emergencies Within the Adult Care Home Sector: A Mixed Methods Case Study

**DOI:** 10.3389/fpubh.2020.541885

**Published:** 2020-12-09

**Authors:** Bhavik G. Bhanderi, Sue Palmer Hill

**Affiliations:** Innovation and Research, Northamptonshire Healthcare NHS Foundation Trust, Berrywood Hospital, Northampton, United Kingdom

**Keywords:** choking, foreign body airway obstruction (FBAO), airway clearance devices, choking algorithm, resuscitation—methods

## Abstract

Foreign Body Airway Obstruction (FBAO) is a medical emergency that can result in death, particularly if swift, appropriate action is not taken. It can be a frightening experience for all involved. DeChoker, an Airway Clearance Device (ACD), might provide an additional alternative in the management of choking; however, limited evidence around its safety and effectiveness makes adoption controversial.

**Objectives:** An independent evaluation to explore the experiences of health and care professionals who used DeChoker in real-life adult choking emergencies, focusing on the product's safety, efficacy and ease of application.

**Design:** Retrospective mixed methods case study, with multiple embedded units of analysis.

**Setting:** UK adult care homes.

**Participants:** Twenty seven incidents of adult choking emergencies self-reported by care home staff where DeChoker was used. This data was augmented by an in-depth exploration of four individual choking incidences.

**Results:** The choking victim's ages range: 45 to 101 years (mean 79.8 years). The device was reported to have successfully removed the obstruction in 26 of 27 cases, with very few complications or adverse events reported. In 21 of 27 incidents (78%) the victim was not required to visit Accident and Emergency. Qualitative data indicated nursing home staff found the DeChoker easy to use and valued its presence as an adjunct to current guideline procedures.

**Conclusion:** There is a dearth of evidence surrounding the management of choking and little innovation in this area for five decades. This retrospective evaluation contributes to discussion regarding the role ACDs might play in the management of choking, particularly in cases where current choking management guidance are ineffective, insufficient, inappropriate or impractical (e.g., frail or movement restricted people). The interview data presents a view from care home staff that the DeChoker, as an ACD, contributed to saving the life of choking victims.

## Introduction and Background

### Foreign Body Airway Obstruction (FBAO)

**Figure 1:**
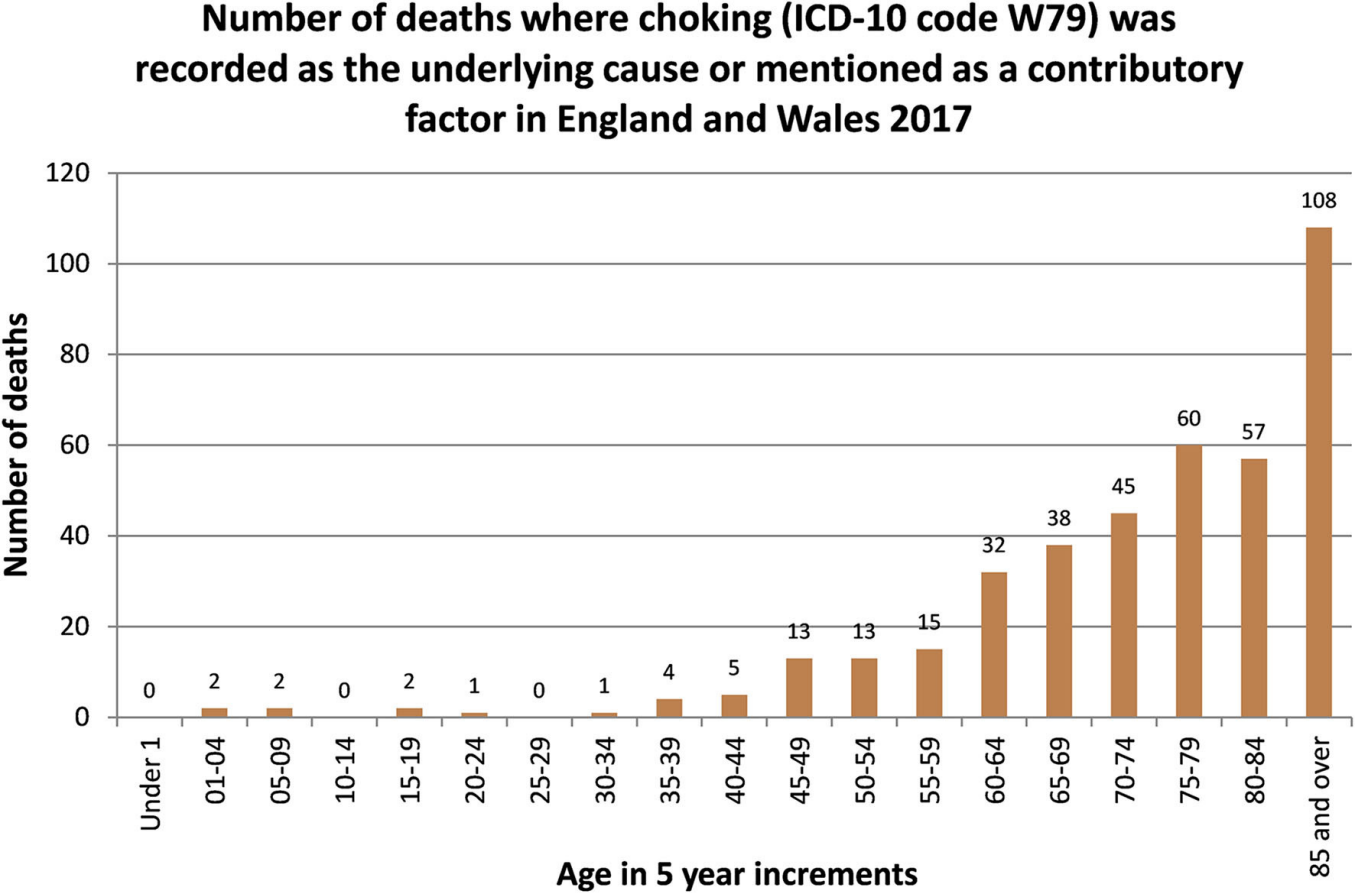
Office of National Statistics.

Foreign body airway obstruction (FBAO), commonly referred to as choking, is a life-threatening emergency, recorded as the underlying cause or a contributory factor in approximately 400 deaths annually in England and Wales (1). It is acknowledged that data on the incidence of choking is largely retrospective and anecdotal and might therefore be under diagnosed and under reported (2–4). The death rate associated with choking is significantly and positively associated with aging (see Figure 1) have also reported choking as the second highest preventable cause of death in this cohort (1, 5, 6). This is due to several factors including, physiological changes to the body's musculature, poor dentition, dementia and other neurological comorbidities, which combined are estimated to increase the risk of choking seven-fold in people over the age of 65 (7, 8). Another significant characteristic of choking is the high percentage of incidences caused by soft foods relative to large solid pieces, in contrast to younger populations (9). This has led to data suggesting where life threatening choking occurs in the elderly population, the mortality rate is over 50% (5).

### Managing the Choking Adult

The scientific evidence for the treatment of choking was first reviewed by the International Liaison Committee on Resuscitation (ILCOR) in 2005, with subsequent reviews by the European Resuscitation Council (ERC) in 2010 and 2015 (10). In the United Kingdom (UK) the ERC guidance informs Resuscitation Council UK guidance on the management and resuscitation of the choking patient, updated in line with the ERC in 2015 (2, 11–13). The sequence of steps for managing choking in an adult is presented in Table 1.

**Table 1:** Sequence of steps for managing the adult victim who is choking. Resuscitation Council UK (2015).

SEQUENCE	Technical description.
SUSPECT CHOKING	Be alert to choking particularly if victim is eating.
ENCOURAGE TO COUGH	Instruct victim to cough.
GIVE BACK BLOWS	If cough becomes ineffective give up to 5 back blows Stand to the side and slightly behind the victim Support the chest with one hand and lean the victim well forwards so that when the obstructing object is dislodged it comes out of the mouth rather than goes further down the airway Give five sharp blows between the shoulder blades with the heel of your other hand.
GIVE ABDOMINAL THRUSTS	If back blows are ineffective give up to 5 abdominal thrusts Stand behind the victim and put both arms round the upper part of the abdomen Lean the victim forwards Clench your fist and place it between the umbilicus (navel) and the ribcage Grasp this hand with your other hand and pull sharply inwards and upwards Repeat up to five times If the obstruction is still not relieved, continue alternating five back blows with five abdominal thrusts.
START CPR	Start CPR if the victim becomes unresponsive Support the victim carefully to the ground Immediately activate the ambulance service Begin CPR with chest compressions.

The evidence base underpinning this guidance for the management of the choking adult has changed little over the last five decades since Dr. Henry Heimlich first recommended abdominal thrusts to expel any foreign body causing airway obstruction (14). There is limited empirical evidence regarding the order of implementation or frequency that these interventions should be carried out, with some evidence that the pressures required to remove an airtight FBAO is far higher than can be achieved by any method individually (3). A recent systematic review was unable to reliably compare the effectiveness of interventions (15). It is recognized that these interventions are themselves not without risk (15–17), particularly when carried out by a lay person in the elderly population (4). This has led some countries excluding the abdominal thrust procedure completely from their choking guidelines (18), recommending instead only back slaps and chest thrusts and a call for safer approaches to foreign body removal to be explored (3).

### Airway Clearance Devices (ACDs)

**Figure 2:**
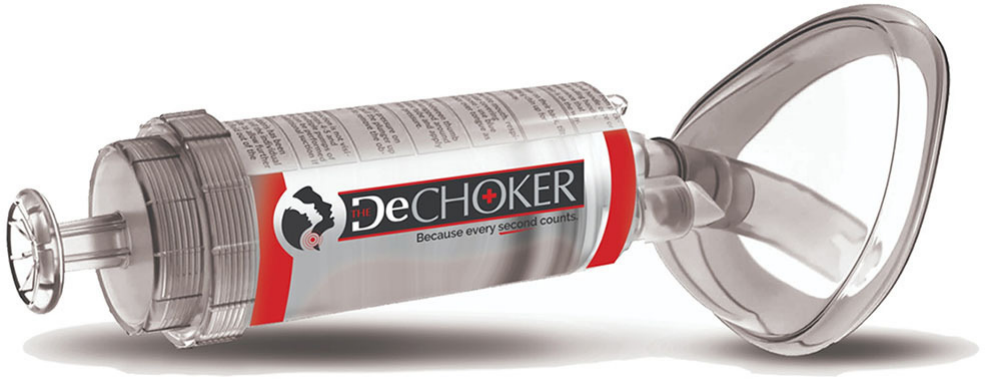
DeChoker device.

**Figure 3:**
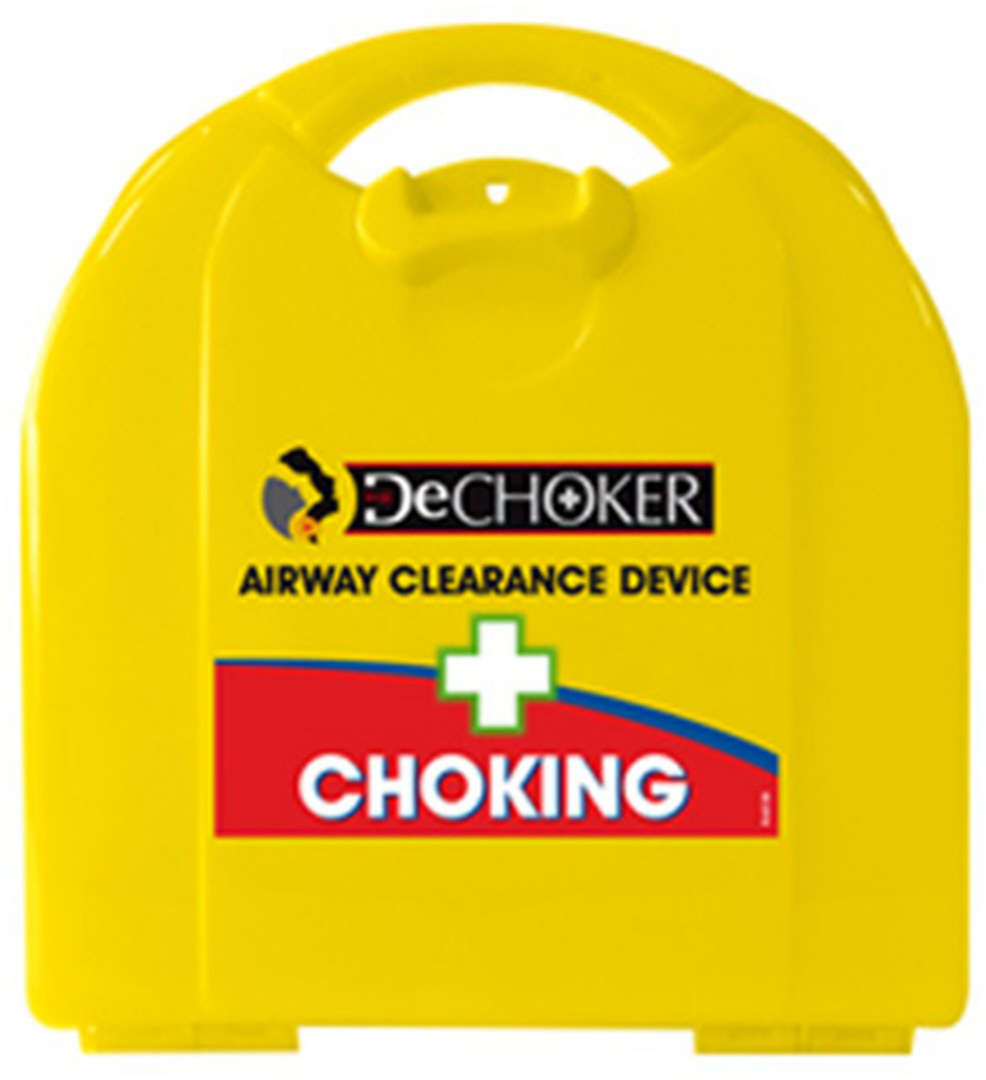
Case with integrated wall-mount bracket.

An Airway Clearance Device (ACD), such as the DeChoker (Figures 2, 3) offer a portable, user powered, hand-held suction device for use in a life-threatening choking situation. It is designed with a one-way valve to prevent air being returned into the trachea and exacerbating any FBAO. The authors acknowledge other ACDs are available on the market, however the purpose of this independent evaluation seeks to explore the design, function and outcomes of the DeChoker device. Cadaver simulations and observational literature suggest the potential of ACDs to support the safe and effective resuscitation of a choking victim (19, 20). Limited published evidence into their effectiveness in a real-world setting however, has made their use questionable. Systematic reviews of the management of FBAO identifies the paucity of robust evidence in this area and the need for further research on the benefits and harms of suction-based airways clearance devices (15, 21). It is recommended that research should detail key demographics, the intervention provided and by whom, as well as outcomes.

### Professional Concerns

Since DeChoker was introduced to the UK in 2017 awareness of the product has grown amongst health and care professionals. Discussions have taken place within these communities of practice, with professionals seeking advice from colleagues and their professional bodies. The Resuscitation Council UK issued a statement regarding the generic use of ACD devices in the UK (22).

“*There is insufficient evidence on the safety or effectiveness of these devices for us to recommend their use, and we are concerned that the use of these devices could delay established treatments for choking; the Council therefore does not support their use.”*

This statement has raised professional concerns amongst individual clinicians regarding deviation from existing guidelines for managing the adult choking patient and Basic Life Support (BLS) protocols, as well as the use of ACDs and any potential associated risks. These concerns were raised by and discussed in the Expert Advisory Group prior to evaluation starting and included the risk of delayed resuscitation, or that resuscitation would not be initiated at all as the ACDs would be used as the first line of treatment. In addition, the lack of anatomically correct simulations raises questions regarding whether the force of the suction may aspirate stomach content and cause an aspiration pneumonia post intervention, or another trauma.

### Post Market Clinical Follow-Up Study (PMCF)

The Resuscitation Council UK however, recognizes the importance developing the clinical evidence on the use of ACDs (22).

“*[The Resuscitation] Council recommends new airway clearance devices should only be used by trained healthcare professionals as part of a formal evaluation.”*

**Figure 4:**
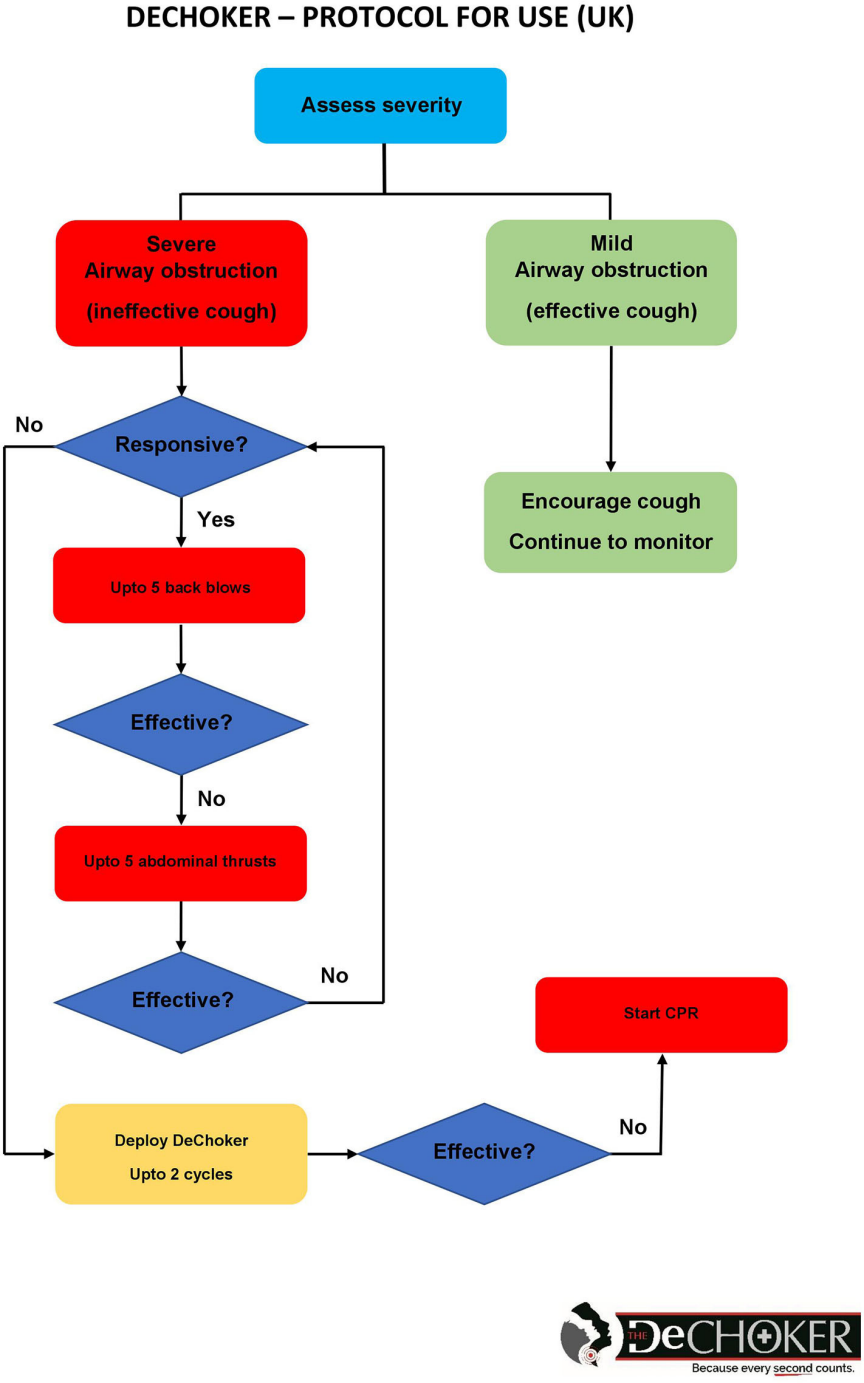
DeChoker protocol for use (UK).

DeChoker is an Airway Clearance Device (ACD) registered with the United States (US) Food and Drug Administration (FDA) for use in a choking emergency. DeChoker was introduced in the UK in late 2017 after obtaining European Conformity (CE) accreditation and is registered with the UK's Medicines & Healthcare Products Regulatory Agency (MHRA) as a Class I medical device. As a “novel” product, guidance from the MHRA advised that introduction should be subject to a Post-Market Clinical Follow-Up (PMCF) protocol. As a significant number of choking deaths are associated with the elderly population, the devices are primarily positioned within the adult care and nursing home sector, supported by a Protocol for Use (PFU) (Figure 4). This, in addition to a training package available for care home staff, sets out how the device should be deployed in a choking emergency, when the guidelines for the management of a choking victim have been initiated but the procedures have failed. When the DeChoker has been used, the care provider is requested to capture and report the incident via PMCF feedback form. The number of devices nationally currently located within this setting is now several thousand, within hundreds of individual services. The first usage was recorded after six months, however, as more devices were situated, the number of deployments has increased with multiple successful usages now reported. This provides the opportunity to study the use of DeChoker within a real-world situation and thus contribute to the developing evidence base of ACDs.

## Methodology

A key issue in any exploration of FBAO are the ethical concerns around control, thus development of the evidence base relies heavily on case study methodology (3). Case study methodology has a long history of use across a range of disciplines, and has established itself as useful as a realistic research approach (23). This approach is increasingly popular within healthcare where it can accommodate multi-level, complex system exploration, and evaluation (24).

### Situating the Research and the Researcher

This descriptive case study explores the use of the DeChoker device within the adult care home sector. The term “*care home”* is used here as a generic term to include both residential and nursing homes, understanding that residential homes provide support with personal care by staff who may not be clinically qualified but that within a nursing home there will always be one or more qualified nurses on duty to provide nursing care at any time (25). The researcher undertaking the evaluation was a healthcare professional with a dental qualification, trained in intermediate life support.

The research and researcher were supported by the Innovation and Research team within Northamptonshire Healthcare NHS Foundation Trust (NHFT) and a multi-professional expert advisory group, whose membership included a Resuscitation Training Officer, Consultant Anesthetist, Consultant in Old Age Psychiatry, Speech and Language Therapist (SaLT), nurses, paramedic teaching staff and NHS Managers. This expert advisory group was drawn from across the wider health and care economy and provided independent critical feedback throughout the conceptualization and protocol design stage of the project, the data collection and analysis and the report writing process, thus supporting the external validity of the case study approach (23).

In keeping with case study design a mixed methods approach was used (26), which included both quantitative and qualitative data, augmented by other available data sources. The case study evaluation provided an holistic understanding of impact and included four embedded units of analysis (23), cases of individual incidences of DeChoker use within the care home setting, explored in depth to provide a granular level of detail and support comparisons across several cases. All data was analyzed through a process of triangulation by the research team (23), supported by the expert advisory group. In taking this approach the study sought to investigate a contemporary phenomenon (the use of DeChoker in a choking emergency) and generate an in-depth, multi-faceted understanding within a real-world context.

### Components of the Case Study Design

A literature review was undertaken by specialist library services to inform the protocol development and the subsequent analysis and reporting of the study. The Post Marketing Clinical Follow-up study systematically collects information on individual incidents of use via self-reported feedback forms. Twenty seven PMCF forms were reviewed in this analysis, containing both quantitative and qualitative data.

The most recent incidences were identified and the care homes were then invited to participate in the in-depth qualitative interviews. Three care homes agreed to participate; a fourth care home declined, feeling that the incident was too distant for accurate staff recall, exacerbated by staff changes. A site visit was arranged and staff recollections of the incident and DeChoker use were captured through semi-structured interviews. The interview schedule is outlined in Table 2. The data captured was explored within each care home setting, including a review of the incident report book. All three care homes willing to be involved in the project were rated “Good” by the Care Quality Commission.

**Table 2:** Interview schedule.

• Can you tell me your job role within the care home and a brief history of your background in health and social care? • How did you hear about DeChoker? • Why did you decide to have DeChoker in the home? • Environment—where is the DeChoker device kept? How far is this from where the incident occurred? • What training was provided by DeChoker UK Ltd.? How did you find the training in terms of usefulness, application, and attention to detail? • What level of life support training is completed by staff in the care home? • Can you describe the choking emergency that took place on [date]? ° How many people were involved in the incident? ° What happened in the events leading up to the choking incident at the care home? ° How many cycles was the DeChoker used? ° Do you know what the obstruction was? ° How safe and effective do you think DeChoker was in the management of this incident? ° How the decision made to use the device? ° Were there any injuries caused? ° Did the resident have dentures? • Was the resident examined by a healthcare professional after the incident? • In the days/weeks following the incident, did the resident develop a chest infection or aspiration pneumonia? • What was your experience of using the device? How did you feel afterwards? • What would you have done if the device was not available to you? • Given the setting that you work in, why do you think DeChoker and airway clearance devices in general is something worth researching?

### Inclusion and Exclusion Criteria

#### Inclusion Criteria

- Care home management and staff involved in choking incident completed and returned PMCF form
- Care home management and staff consent to allow the research team access to site
- Care home staff able and willing to participate in semi-structured interviews
- Care home consent to allow the research team access to incident report book/system.

#### Exclusion Criteria

- Care home lack of consent to participate
- Care home staff did not consent to participate.

### Data Analysis

Initial reduction and display of data was undertaken as preliminary analysis strategy by tabulation of data from the PMCF forms (27). Each semi-structured interview was reviewed and analyzed using the process of thematic analysis outlined by Braun and Clarke (28). Comparisons were then made across the interview data, and triangulated with other data. This process took place before, during, and after data collection in several iterative cycles, informed by theoretical propositions of the literature review (29). The Expert Advisory Group oversaw the analysis and report writing process to help ensure the trustworthiness (i.e., credibility, transferability, dependability, and confirmability) of the results (30).

### Duration

The PMCF forms were collected over a period of 18 months and the individual incidences explored took place within the last 12 months. The case study evaluation including the semi-structured interviews took place between September and December 2019.

### Ethics

A governance review of the evaluation was carried out and as this was a retrospective evaluation of care home staff perspectives and experiences, NHS Research Ethics Committee review was not required. All care homes were informed of the evaluation and consented to participate, with informed consent taken from individual staff members. All data was fully anonymised before being shared with the research team, supported by a data sharing agreement.

### Patient and Public Involvement

No patient involved.

### Findings

#### Post Market Clinical Follow-Up (PMCF) Forms

In December 2019, 27 post market clinical follow-up (PMCF) forms had been completed and were included in the analysis. The residents were aged between 45 and 101 years (mean = 79.8, mode = 86, median = 84). All “*type of obstructions*” were recorded as food, with consistency varying between porridge and a solid piece of meat. The quantitative data from these forms are displayed in Table 3.

**Table 3:** Quantitative data from PMCF forms.

	**Yes**	**No**	
Had the user received training in device usage?	23	4	
Were back blows given?	19	8	
Were abdominal thrusts given?	11	16	
Was the patient unresponsive when the device was used?	9	18	
Did the device successfully remove the obstruction?	26	1	
Was the patient seen by a health professional post-incident?	23	4	
Was the patient taken to A&E?	6	21	
Did the patient remain in hospital after the incident?	2	4	
	**Standing**	**Sitting**	**Lying down**
What was the position of the patient when the device was used?	2	19	6

The device successfully removed the obstruction in 26 of the 27 cases; the remaining incident stated that although the device had not successfully removed the obstruction, the resident had managed to cough it out, with the device helping to remove excess of phlegm, allowing the resident to breathe and reduce risk of aspiration. In 16 cases abdominal thrusts were not attempted, the reports stated that these could not be attempted due to the position of the resident, with further information stated that residents were seated in a wheelchair or sat/lying in bed. The oldest resident was reported to be 101 years; special reference was made to this in the comment.

“*Back blows and abdominal thrusts were not attempted as this would be inappropriate due to the age and frailty of the person. Also profiled chair that person was sitting on would be an obstruction for the staff”*

Twenty-one residents (78%) did not require a visit to Accident and Emergency and of the six that were taken to hospital; four were discharged on the same day with only two remaining in hospital specifically for their care packages to be updated. Four forms stated that the individual using the device had not received formal training, but of these, three added that they had read the information leaflet and directions for use.

### Interview Analysis

Four individual incident reviews, within three care homes in England were explored in-depth and a total of five staff were interviewed. Two of the five staff members interviewed had previously witnessed death by choking. The following themes were identified.

#### Safety and Effectiveness

All reviews reported a successful outcome with the obstruction cleared from the airway. The residents were monitored for complications post-incident and were not observed to develop aspiration pneumonia or a chest infection. The decision to use the DeChoker device was made due to ineffective clearance of the obstruction through conventional methods of back blows and abdominal thrusts. The type of obstruction could also have a bearing on this.

“*In my past experience before, I've always managed to dislodge whatever it is with good back slaps. But this was the first time I've not seen that work. But as I say, it could be because it was porridge.”*

In all four reviews, the resident had lost consciousness before the device was used, and in most cases consciousness appeared to be regained immediately after the obstruction was removed. In one incident, however, the resident regained consciousness only after the ambulance crew arrived. In this instance the obstruction was caused by a piece of roast potato that was not fully removed by the DeChoker. In another situation the interviewee described the resident choking on two different types of food simultaneously; the DeChoker effectively removed the top item, which allowed the deeper obstruction to dislodge. On this occasion a call to emergency services had already been made, but when the obstruction was removed the ambulance was canceled.

Complications, including mouth trauma were explored within the interviews, in two episodes small amounts of bleeding from the mouth were reported. In one of these incidents the interviewee had experienced difficulty positioning the DeChoker because the resident had “*locked her jaw shut.”* The participant also reported:

“*Initially I couldn't get the device past her teeth. So we had to as much as we can and as gently as we could pry her teeth open and it was really difficult. But eventually we managed to get it in and used the device twice.'…………. ‘The staff had reported there was a little bit of bleeding in the mouth. And I said, well I'm not surprised because we had to put the device in her mouth with her clamping her teeth*.”

In a second incident where an injury was reported staff reported that the victim had bitten their tongue as they started to choke. The presence of dentures was identified as an additional complication in this case; staff felt to ascertain a causal relationship between the Dechoker, dentures, and a bitten tongue was problematic. The presence of dentures and how to manage these in a choking situation was also reported in two other reviews, with staff unclear whether to leave them in or take them out, in one instance a mixed approach was used. Injury was a subject mentioned by all interviewees, with interviewees articulating that incorrect delivery of abdominal thrusts could also exacerbate an injury.

“*My feeling is the level of risk [of injury] using the DeChoker is probably a lot less. My fear is if a member of staff does not do abdominal thrusts properly then somebody is going to get seriously injured”*

In summary all participants felt that the DeChoker had value, acting as an additional lifesaving resource when back blows and abdominal thrusts had failed to remove the obstruction, or couldn't be performed. They reflected that it was reassuring to have it on site (in a similar way to a defibrillator being on site) and that it contributed to saving lives. It was felt that although there was a risk of minor oral injury, this was felt to be acceptable, particularly when the alternative of back slaps and abdominal thrusts was known to have serious risk within this client group (e.g., rib fracture, bruising, internal bleeds).

“*It's the sense of achievement and knowing that you saved somebody's life. Yes it's through a device and it's been made for that reason, but because it was such a positive outcome and because he recovered so quickly, and we've still got him. This chap is in his nineties. Fantastic outcome.”*

#### Product Design

The recognition of the DeChoker as an additional resource contributed to a discussion regarding the product design, with both the simplicity of the device and its ease of use acknowledged.

“*You know, it's not something that you have to read reams and reams of instructions for, for it to actually work. It's just a simple piece of kit that absolutely anybody could use”*

Simplicity, however did not necessarily overcome inexperience; a participant commented although she had completed online training she had not experienced pulling on the device until the incident occurred. It took her by surprise how hard she had to pull, but was able to remove the obstruction.

“*I did find it was quite hard to pump at first, because obviously it was the first time I'd used it, I knew exactly what I needed to do, it's just I didn't realize how strong it would be. So as I was doing it, I did manage to free some stuff from out of her throat”*

The device has a specially designed one-way valve so that air cannot be pushed into the oropharynx. This allows the user to maintain their position and hold of the device whilst simultaneously resetting the device for a second pull. This feature was appreciated by staff using the device, as was the clear chamber that provided a visual confirmation that the obstruction, whole or part, had been removed. One interviewee reinforced the ease of use but commented that they thought a potential challenge might be to get the device placed properly in the victim's mouth. The device requires positioning of the face mask whilst pulling the plunger, and interviewees felt that although it could be managed by a single person, additional team support was beneficial, and that the device needed to be close by so that the casualty was not left unattended.

The device is packaged in a wall mounted medical devices box that can be removed to be transported to where it is needed. Three interviewees did not have any difficulties accessing the device, however, in one incidence a distressed staff member struggled to open the box, which was then swiftly opened by another member of staff.

In summary, the device was identified as being well designed and easy to use, either singularly or as a team, although it was recommended that training was given to anyone who might use it in an emergency choking situation. The features of the one way valve, the clear chamber and the portable storage box were appreciated.

#### Adherence to Protocol

All care homes staff interviewed undertook basic life support within their mandatory training, which included the management of choking. DeChoker provided training both in person and/or via an online training package. Two participants had received training in person, three completed online training and one participant received both forms of training. The participants that completed training in person felt that it benefitted them to practice using the device.

“*[Training] was delivered in person, so we actually used a manikin which was very helpful”*

The online training was considered to be sufficient in content and easy to understand with the time taken to complete ranging between 15–30 min. It was felt that repeating this training more regularly then annually might ensure that it remain at the forefront of people's minds. Back blows and abdominal thrusts were administered in all four incidents explored with the individual deemed unconscious when the device was used. This reflects the DeChoker protocol and demonstrated adherence.

“*Eyes were rolling, his pallor had changed; he was going blue around the lips. We tried to abdominal thrusts and we tried to do back blows. By this point, he was losing consciousness, he'd become a dead weight, he was beginning to slump, so we put him back in the chair”*

In all the incidents care home staff had worked together as a team to manage the choking emergency. This meant that between four and six members of staff were actively involved, either dealing with the emergency itself or tending to other residents in the vicinity. Staff articulated that training and practicing as teams within their workplace meant they were ready to respond to medical emergencies. It was suggested however, that the inclusion of roleplay within DeChoker training, similar to other medical emergencies training, would be useful to ensure an automated response to choking events. This would additionally support the development of leadership and the identification of specific roles in the management of a choking emergency.

“*If you have a five minute role play at the end of each of those, because we are really high risk choking group, our home is high risk, even though we've got measures in place, then it becomes second nature”*

When asked “*what would you do if the device was not available to you*?” all responses stated that they would continue with the basic life support guidelines and administer CPR until the ambulance crew arrived.

#### Management of Different Client Groups

This theme was drawn from the interviewees' reflections of events and consideration of their client group's needs. They all reflected that there was a general lack of tailoring of both procedure and training to the population within care homes. This was particularly relevant when considering basic life support where some protocols might be inappropriate or unachievable for very frail residents, bariatric residents, and/or wheelchair users. The appropriate management of individuals in a wheelchair created a lot of discussion, and was felt to be routinely missed in basic life support training. One interviewee shared their own investigation concluding that there is no clarity in guidance, presenting staff with a significant problem.

“*I did look at a video online, something in America where they was dropping the wheelchair back onto the floor so that they were on their back and then doing the abdominal thrust from that position. And I spoke to our physiotherapists at wheelchair clinic saying “what do you recommend?” and “what training have you had?” And they haven't had any training at all either.”*

It was also noted that a resident could be physically large compared with staff and that staff might sustain an injury trying to move very large or heavy residents to a position to perform resuscitation.

“*I think I have to weigh up the size of my staff against the resident for a start”*

Interviewees felt that in situations with different client groups the DeChoker, could be used in any position and was a “*lifeline”* and suggested that in their client group the outcome of choking without access to a DeChoker could be significantly different. This idea that the DeChoker could be of benefit to specific client groups was extended to a discussion around sedation of residents with challenging behavior, and the high choking risk in these individuals. Although staff identified that the mealtime information sheets, care plans and choking risk assessments that are all intended to reduce the risk, they also commented that there was a lot to juggle at meal time and having the DeChoker on hand could be of additional benefit.

In summary, this was an area that presented a lot of discussion, highlighting that current guidelines did not recognize the needs of this specific patient cohort, presenting increased dangers not only to patients but to staff alike. There was little or no resource to support staff to develop their knowledge in this area; this created doubt and confusion.

#### Reflective Practice

The participants found recalling the events that had taken place emotional, with two participants becoming tearful during the interviews. This expression of emotion continued in the aftermath of the incident, with some team members stating that they experienced distress in the days that followed. This appeared to be related to staff and their personal connection to their individual resident and the impact of the emergency situation.

“*I was a mess. I was an absolute mess….I went and stood outside just looking over the fence and I just thought “what the hell has just happened?” You know, it's the first time I've ever had to deal with anything like that in the 15 years I've worked in care.”*

Three of the interviewees commented that they felt they were on high alert and became overprotective of the individual resident in the days that followed the incident. All participants had been asked by colleagues if they required a debrief and one participant acknowledged the need to have access to psychological support, something not always available in the care home sector.

“*We escalated straight away to a safeguarding incident. So the entire shift was nearly then just went with reports and reports and reports. Doing the debriefing, keep coming out making sure staff were ok.”*

The need to reflect on the lessons learned from the events was also acknowledged. The practical considerations of revising care plans, updating risk assessments and making referrals to SaLT were identified as paramount, although this created additional administration. Mitigating risks once it has been highlighted by an incident should be an integral part of practice. A need to focus on the positive aspects of the experience was also identified.

“*But then when you sit down, we all went into the clinic and everything was settled and sorted, the staff involved and the nurses, we just had a debrief chat. And that's when you start focusing on the positive sides of it. At the end of the day we saved the man's life.”*

This led two of the participants to share their experiences of previous choking incidents, which although had occurred many years previously were still very much in their minds. In both incidences the individual concerned lost their life, and the trauma of these events were obvious.

“*This home has had a gentleman die from choking. Early, probably ten years ago. Maybe a little bit longer.” “I think we're very aware of the risk with choking. And I know we was doing everything and the paramedics, they did come for that gentleman and they worked on him for 20 minutes and there were no way that it was coming out. And I think that I don't think I would've coped with somebody dying, from choking. I think it must be absolutely dreadful. And that helpless feeling. Even though you're doing what you're trained to do on your first aid. I would not want to be in that situation.”*

Two members of staff, however, confessed that after using the DeChoker they felt confused and had doubts about how they handled the situation. One participant ended the interview by commenting how they had sought support from external SaLT colleagues, pointing out that this gave conflicting information that added to their anxiety.

“*I would like to share that following the incident I did phone, I spoke to the speech and language therapist because the referral had gone through to them to do another assessment on [name redacted], and said that we'd used the DeChoker. And she did say that they don't support the DeChoker in the NHS. So then you're thinking, you know “are we using a piece of equipment that we shouldn't really be using? It's an American piece of equipment” Do you know what I mean? I find it quite reassuring that you're actually coming in now and researching whether it will be of benefit or not. We've certainly found it a benefit.”*

In summary for staff involved with or witness to an emergency choking situation, this was found to be a highly traumatic and stressful situation. Even a positive outcome resulted in a stress response, and a negative outcome stayed with an individual for many years. This has the potential to impact on their ability to undertake their roles. Although in these cases the outcomes were all successful, not having clear guidelines and professional support for the use of DeChoker was a cause of concern and anxiety to care home staff.

## Discussion

The majority of the population were elderly, with qualitative data from both the PMCF forms and interviews indicating a high degree of comorbidity and frailty. This links to the literature that identifies a significant risk of choking in the elderly; (1) given the aging population of the UK this will become an increasingly important issue. Five themes were generated from the qualitative data:

- Safety and effectiveness
- Product design
- Adherence to protocol
- Management of different client groups
- Reflective practice.

In the incidences reported the majority were not required to attend hospital, although 85.1% of victims were reviewed by a health care professional within the care home. In the incidences explored in depth no resident was reported to have residual aspiration pneumonia. Although in two incidences mouth trauma was identified, neither could be linked directly to the use of the device and staff felt this trauma to be minor in comparison to choking itself or other resuscitation interventions that might have been utilized. In general staff felt that the DeChoker was easy to use and well designed, with the non-return valve and the clear chamber particularly useful features. They felt that the figure for accident and emergency attendance, fatality or post incident sequela might have been significantly higher if the DeChoker had not been deployed.

Training in the use of Dechoker was felt to be adequate, however it was suggested this could be improved through role play scenarios to strengthen the staff response. The Dechoker “Protocol For Use” clearly states that Resuscitation Council UK guidance should be followed (2), with the victim being unresponsive prior to the DeChoker being deployed. The PMCF data indicated that this protocol was not strictly adhered to as: 29.6% (*n* = 8) of victims did not receive back blows; 59.6% (*n* = 16) did not receive abdominal thrust; and 66.7% (*n* = 18) of victims were responsive when the device was deployed. However, descriptions were given regarding the high level of clinical judgement used by very experienced staff, coupled with their detailed knowledge of their residents. Staff felt able to both determine the severity of the incident and the risk to the patient of administering the primary choking management techniques in elderly frail residents. This clinical risk around choking management techniques especially in elderly patients are well documented (16–18, 31). Many of the staff interviewed reflected on the lack of specific guidelines for their client group and the uncertainties this creates. The expert advisory group reflected on this but also acknowledged that basic life support training may not be standardized across care homes, and that this might account for some of the variation in the data.

Care home staff also raised the issue of the management of dentures in a choking emergency. It is unclear from staff accounts whether trauma to oral soft tissues were caused by ill-fitting dentures or the device. Complete dentures are likely to be more challenging than partial dentures, as a complete denture requires a degree of proprioception and muscular control to aid retention and stability, particularly for a lower denture; unconsciousness would render this mechanism to fail. There is a lack of recommendation about dentures within current resuscitation or choking guidance, presenting a challenge to staff dealing with these situations.

Similar anxiety was articulated for the resuscitation of the bariatric or wheelchair resident, although staff had undertaken their own research, they identified no management solution that did not put individuals at additional risk. The ability to use the DeChoker in any position was thought valuable; of the 27 incidences report on, 70.4% (*n* = 19) of victims were in the seated position and a further 22.3% (*n* = 6) of victims in the lying position. In these examples the qualitative data suggested that moving the victim to perform standard chocking management interventions would not have been practically possible.

The experience of watching a victim choke was identified by staff as traumatic; they articulated that had the outcome of events been negative, it might impact on their ability to continue in their jobs. Well trained experienced care home staff are a valuable commodity; interventions that support staff in the delivery of care, whilst addressing their safety and wellbeing needs should be a high priority. This should extend to the development of clear professional guidance on whether the DeChoker should be used in choking emergencies to prevent confusion and anxiety.

## Study Limitations, Areas of Further Study and Development

This study was a retrospective case study that reviewed a small number of reported choking emergencies where a DeChoker had been used, with an in-depth exploration of four incidences in three care homes carried out over a short time period. All the care homes made a positive choice to participate, the self-selecting bias of this approach is acknowledged. In addition case study methodology has been criticized within healthcare literature for its lack of rigor, however the nature of choking makes other methodologies problematic. The approach employed was supported by the expert advisory group to mitigate these issues.

The expert advisory group noted concerns around the accuracy of recall of events by care home staff, especially given the time lag, noting that in several cases events had occurred several months previously. They also questioned the accuracy of staff assessment of the victim's state of consciousness in these emergency situations, and acknowledged there was no way of assessing the quality of any back blows and abdominal thrusts given. The observational nature of the data is acknowledged.

The impact and influence of professional bodies and guidance on innovation evaluation and adoption should also be noted here. The expert advisory group had representation from the outset from a range of professions. One representative however discussed the DeChoker project with colleagues from their team and their professional body. After these discussions the team made a collective decision for their representative to leave the Expert Advisory Group, and their profession's view was therefore not represented in the final discussion.

In the care home setting, continued collection of the PMCF form data will be a key aspect of developing the evidence base. This should be augmented by further qualitative evaluation of the experiences of using the device. One question that needs to remain under review is the issue of mouth trauma; in this small sample the findings were inconclusive. A second specific question requiring addressing is the responsiveness of the victim at the time of device deployment. A high proportion of forms indicated that the victims were responsive; this is contra to the evidence of the in-depth exploration and warrants further investigation.

## Conclusion

A choking emergency is traumatic and distressing for victims and health care staff alike; however the treatment and supporting evidence base for the management of the adult choking victim has developed little over the last few decades. The risks associated with administering the current interventions for the management of the adult choking victim for patients and staff are recognized. ACD application might improve the safety of management, particularly within a growing elderly population.

DeChoker is an innovative product that is available for use within the care home sector. The evidence from this evaluation demonstrated that it is being used effectively and safely in emergency choking situations where recommended management methods have failed or cannot be implemented. It appears to be valued as an adjunct resource by the care home staff that used it. Training on the DeChoker device was seen as adequate, but could be further strengthened by regular refreshers and role play scenarios as used in other Basic Life Support training.

Clearer guidance is required for health and care professionals around the resuscitation of the elderly or immobile patient. Specific guidance for the resuscitation of this population does not exist, but the issues are recognized by those that care for them. This should include guidance on the management of dentures, bariatric, wheelchair, and bedbound patients. Further evaluation and research would be beneficial to develop a more robust evidence base that can support ACD adoption into resuscitation guidelines.

## Data Availability Statement

The datasets generated for this study are available on request to the corresponding author.

## Ethics Statement

Ethical review and approval was not required for the study on human participants in accordance with the local legislation and institutional requirements. The participants provided their written informed consent to participate in this study.

## Author Contributions

BB: principal investigator, setup and delivery, governance, study design, data collection, analysis, write-up. SP: governance, study design, write-up. All authors contributed to the article and approved the submitted version.

## Conflict of Interest

The authors declare that the research was conducted in the absence of any commercial or financial relationships that could be construed as a potential conflict of interest.
